# Neurogenic diabetes insipidus in a critical patient with COVID-19 pneumonia in treatment with extracorporeal membrane oxygenation: a case report

**DOI:** 10.1051/ject/2023021

**Published:** 2023-09-08

**Authors:** Bruno Samaniego-Segovia, Lilia Rizo-Topete, Montserrat de la Garza-Gomez, Cesar Alejandro Rodriguez-Salinas, Salim Martínez-Cadena, Alicia López-Romo, Rene Gomez-Gutierrez, Uriel Chavarría-Martínez, Sergio Sánchez-Salazar

**Affiliations:** 1 Internal Medicine Residents, Department of Health Sciences, Christus Muguerza Health System, UDEM 64060 Monterrey Nuevo León México; 2 Nephrology of the Critically Ill Patient, Internal Medicine, Internal Medicine Professor, Department of Health Sciences, Christus Muguerza Health System, UDEM 64060 Monterrey Nuevo León México; 3 Associate Professor of Nephrology Service, Hospital Universitario “José Eleuterio González”, UANL 64460 Monterrey Nuevo León México; 4 Infectious Diseases, Internal Medicine, Internal Medicine Assistant Professor, Department of Health Sciences, Christus Muguerza Health System, UDEM 64060 Monterrey Nuevo León México; 5 Pediatrics, Director of the ECMO Unit, Christus Muguerza Health System, UDEM 64060 Monterrey Nuevo León México; 6 Pulmonary and Critical Medicine, Internal Medicine Professor, Department of Health Sciences, Christus Muguerza Health System, UDEM 64060 Monterrey Nuevo León México; 7 Pulmonary and Critical Medicine Professor, Hospital Universitario “Dr. José Eleuterio González”, UANL 64460 Monterrey Nuevo León México

**Keywords:** Extracorporeal Membrane Oxygenation, COVID-19, Diabetes insipidus, Neurogenic, Respiratory distress syndrome

## Abstract

The following case report analyses a patient with extracorporeal membrane oxygenation (ECMO), who suffered from a severe Acute Respiratory Distress Syndrome (ARDS) due to COVID-19 pneumonia. ARDS is defined as a diffuse and inflammatory injury of the lungs; classifying this as severe when the ratio of arterial oxygen tension to a fraction of inspired oxygen (PaO_2_/FiO_2_) is equal to or lower than 100 mmHg. To decide if the patient was suitable for the use of ECMO therapy, the ELSO criteria were used; and in this case, the patient matched with the criteria of hypoxemic respiratory failure (with a PaO_2_/FiO_2_ < 80 mmHg) after optimal medical management, including, in the absence of contraindications, a trial of prone positioning. During hospitalization, the patient presented a Central Diabetes Insipidus (CDI), probably explained by the damage hypoxia generated on the central nervous system. There are few reports of this complication produced by COVID-19. The case is about a 39-year-old woman, who started with ECMO 6 days after the beginning of Invasive Mechanical Ventilation (IMV), because of a severe ARDS. On the fifth day of ECMO, the patient started with a polyuria of 7 L in 24 h. A series of paraclinical studies were made, but no evidence of central nervous system lesions was found. After treatment with desmopressin was initiated and the ARDS was solved, polyuria stopped; with this, CDI was diagnosed. There are many complications secondary to the evolution of COVID-19 infection, and some of them are not yet well explained.

## Overview

The COVID-19 pandemic had a great epidemiological impact, affecting almost 130 million persons and with about 2.8 million deaths around the world during the first trimester of 2021 [1]. The SARS-CoV-2 virus has a high affinity to the receptor of the Angiotensin Converting Enzyme 2 (ACE2), which explains its wide clinical spectrum and the complications that affect a huge amount of body systems [2]. Acute Respiratory Distress Syndrome (ARDS) can be generated because of this virus. ARDS is defined as a diffuse and inflammatory injury of the lungs; classifying this as severe when the ratio of arterial oxygen tension to a fraction of inspired oxygen (PaO_2_/FiO_2_) is equal to or lower than 100 mmHg. Also, there are multiple complications related to this clinical scenario. Cardiopulmonary affections are related to a greater mortality of the critical patient [3]; but also, there are endocrinologic complications that increase the morbidity [4]. One of those complications that can be found in this type of patient, is Central Diabetes Insipidus (CDI); which is a disorder that’s characterized by the excretion of large amounts of hypotonic urine, explained usually by deficient synthesis of arginine vasopressin (AVP) in the hypothalamic-neurohypophyseal system. CDI is a form of polyuria-polydipsia syndrome that makes excessive hypotonic polyuria (>50 mL/kg body weight/24 h) and polydipsia (>3 L/day). In this clinical case, the two main differential diagnoses were Central and Nephrogenic Diabetes Insipidus; and for this reason, urinary electrolytes and osmolality were taken. Treatment for CDI is primarily aimed at decreasing the urine output, usually by increasing the activity of the vasopressin hormone; and the correct replacement of fluid losses. The following case describes a 39-year-old woman who develops CDI during her hospitalization, due to Acute Respiratory Distress Syndrome (ARDS) from COVID-19 pneumonia, associated with the use of Extracorporeal Membrane Oxygenation (ECMO).

## Clinical case description

**Figure 1:**
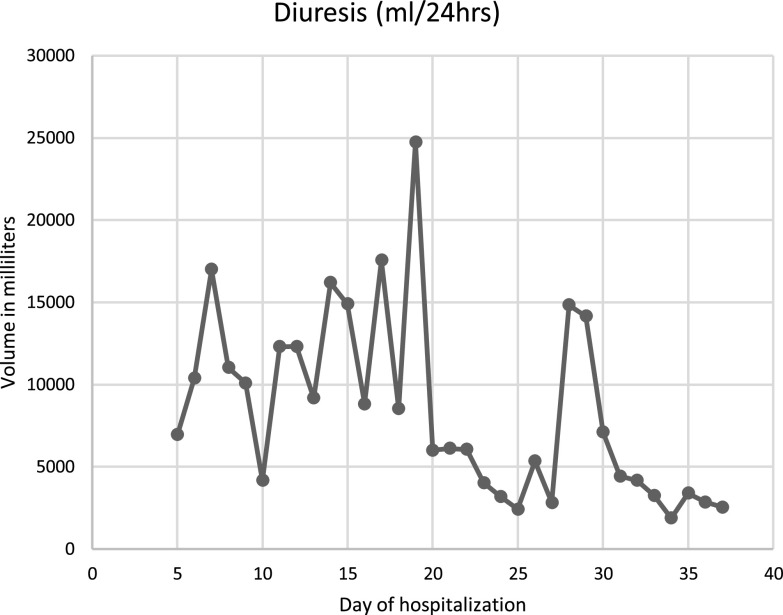
Graph 1: Diuresis per hospitalization day.

**Figure 2:**
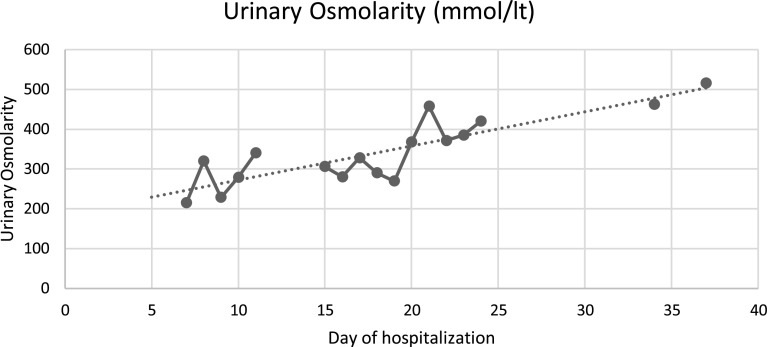
Graph 2: Urinary osmolarity per hospitalization day.

**Figure 3:**
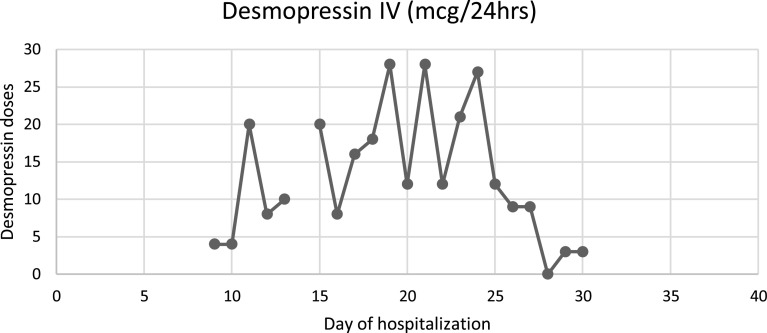
Graph 3: Desmopressin doses given by hospitalization day.

The case is about a 39-year-old female, without any relevant medical background; which started 29 days prior to her admission, with insidious and progressive generalized myalgias and arthralgias; followed by non-productive, intermittent cough; to which dyspnea on small efforts was added, associated with intermittent febrile peaks. The diagnosis of COVID-19 pneumonia was confirmed, and for this reason, she was admitted to another hospital, 19 days prior to her admission to our hospital; where she presented a torpid evolution and a need for oxygen therapy with progressive requirements, starting invasive mechanical ventilation 6 days prior to our hospital admission. Also, computed tomography was realized, showing interstitial pneumonia with a ground glass pattern. However, during this hospitalization, she continued with hypoxemia refractory to changes in position and FiO_2_ at 100%; because of this, she was referred to our hospital to start with ECMO therapy. Once the patient arrived at our hospital, it was determined that it met the criteria for ECMO support, which according to the ELSO criteria [5], was the presence of hypoxemic respiratory failure (with a PaO_2_/FiO_2_ < 80 mmHg) after optimal medical management, including, in the absence of contraindications, a trial of prone positioning. At her arrival, some of the parametric measurements were a height of 1.87 m, a weight of 86 kg, body weight index of 24.6 kg/m^2^; also, the vital signs were a heart rate of 67 bpm, respiratory rate of 34 rpm, arterial blood pressure of 113/78 mmHg and a temperature of 36 °C. Analgesia and sedation were optimized once the patient was established in the Intensive Care Unit (ICU) of our hospital. Also, protocol laboratories such as blood cultures were taken, initiating prophylactic antibiotic therapy with Meropenem 2 g every 8 h, linezolid 600 mg every 12 h and Voriconazole 300 mg every 12 h. Once admitted to the ICU, the ventilator was adjusted to the modality of Bilevel, with a respiratory rate of 14 rpm, PEEP high of 20 cmH_2_O, and PEEP low of 10 cmH_2_O. The initial venous gases with these ventilator parameters were a pH of 7.51, pCO_2_ 49, bicarbonate 39.1, Lactate 1.9, and oxygen saturation of 89%; and no arterial gases were taken. Hours after her arrival at the hospital, the patient was cannulated to ECMO, with a V-V ECMO type; and a cannulation strategy of one femoral cannula that led to the superior vena cava and one jugular cannula that led to the right atrium; because this strategy was used for oxygenation support (as no ventricular support was needed). Once the ECMO therapy started, 3 days later, elective extubation was realized and high flux cannulas were installed with a flux of 60 L/min and FiO_2_ of 50%, with a peripheral oxygen saturation of 95%. Although, on the fifth day of hospitalization, the patient started with a sudden polyuria of 7 L in 24 h and serum sodium (Na) of 145 mmol/L (normal values of 135–145 mmol/L). Because of this, volume repositions of 50% of the milliliters lost in diuresis per hour was made with the Hartmann solution. As the amount of urinary volume continued to increase up to 17 L in 24 h, the levels of urinary osmolarity and sodium were measured, reporting an osmolarity of 215 mOsm/Kg and a urinary sodium of 111 mmol/L. Due to these results, treatment with intravenous vasopressin was initiated, with 30 IU every 24 h. Despite this, the patient persisted for 3 consecutive days with polyuria of 10 L per day; changing intravenous vasopressin to desmopressin. Later, a brain Computed Tomography (CT) was made, with no evidence of pituitary adenoma or vascular event, making the diagnosis of central diabetes insipidus (CDI). The patient received an intravenous dose of 20 mcg/day of desmopressin; but due to persistent polyuria of 12 L in 24 h, 25 mg of intravenous hydrochlorothiazide every 12 h was added, reducing the desmopressin dose to 8 mcg every 24 h. After 15 days of hospitalization, hydrochlorothiazide was discontinued. On her 18th day of hospitalization, polyuria recurred, showing serum Na of 145 mmol/L, urinary Na of 129 mmol/L, and urinary osmolarity of 340 mOsm/kg. Oral indomethacin was started at a dose of 75 mg/day, with a progressive increase in dosage up to 125 mg, continuing this dosage for 12 days in which urinary volumes decreased to 4.5 L/day and IV desmopressin dose to 3 mcg/day, which later was decided to switch to inhaled desmopressin. After this, diuresis improved to 2,800 mL/day, serum Na decreased to 141 mEq/L and urinary osmolarity increased to 519 mOsm/kg on the 37th day of hospitalization. Daily applications of inhaled desmopressin were decreased and on the 40th day, diuresis was less than 2.5 L/day, with urinary osmolarity of 516 mOsm/kg. Treatment was continued with ECMO, vasopressor support, and IMV during this time. While this therapeutical adjustment was made, the ECMO had several changes in the way it ran; as hypovolemia caused by polyuria, modified the extraction pressure, causing a flow decrease. Whenever there was significant hypovolemia, which translated into an increase in extraction, it led to the occurrence of chattering in the extraction line, as a collapsing right ventricle moved this line; also the ECMO marked the presence of obstruction (made by the phenomenon of the collapsing right ventricle, previously described). As soon as this was seen, the ECMO flow was decreased by about 50% and repositions of volume were made. Once the volume was replaced IV, the ECMO flow was again increased to previous levels. Later, as the pulmonary condition improved, ECMO was withdrawn after 55 days of extracorporeal support. Physical rehabilitation was continued, and the supplementary oxygen supply was progressively reduced. Finally, she was discharged on her 84th day of hospitalization, requiring supplementary oxygen through nasal prongs at 2 L per minute. For a summary of diuresis volume per day, urinary osmolarity per day, and desmopressin doses per day, please refer to Graphs 1–3 respectively (Figures 1–3).

## Comment

COVID-19 disease has changed the diagnostic approach to critically ill patients, which has impacted the clinical practice of multiple disciplines [4]. The aggressive behavior of its cardiopulmonary complications represents a therapeutic challenge for intensive care medicine, which has positioned ECMO therapy as a potentially effective alternative for patients with hypoxemia refractory to IMV [6]. Currently, cardiopulmonary, infectious, hematologic, neurovascular, and endocrinologic complications of COVID-19 pneumonia are described; the last one in a smaller proportion [7]. Few cases have been described with hypothalamic-pituitary axis involvement [8–11] since in the reported cases central hypoxia and/or autoimmune alterations are pointed out as possible mechanisms for the development of CDI [9]. For diagnosis, tumor, hemorrhagic or ischemic causes should be ruled out [10, 12], which were not found in our patient. According to the clinical course of the presented case, we assume that prolonged and refractory hypoxemia was associated with the development of this disease. We present a case of CDI in a patient with critical COVID-19 under ECMO therapy, which we believe could explain the severity and difficult management of polyuria, despite the administration of vasopressin, desmopressin, and indomethacin, in this case, presented. In conclusion, despite the few reports of endocrinological complications from COVID-19, central diabetes insipidus is currently described as a medium- and long-term complication. We assume that its presence is secondary to the development of hypoxic encephalopathy in patients with refractory hypoxemia from severe ARDS. Therefore, we alert physicians to recognize hydroelectrolytic and urinary alterations in advance to prevent the development of this potential complication.

## Data Availability

All available data are incorporated into the article.
